# Supplementary material for: Doppler-defined pulmonary hypertension in β-thalassemia major in Kurdistan, Iraq
Source: PLoS One. 2020 Dec 10;15(12):e0243648. doi: 10.1371/journal.pone.0243648 (PMC7728223; doi:10.1371/journal.pone.0243648)
Supplement: Attachment — Submitted filename: PONE-D-20-17752_reviewer.pdf [file pone.0243648.s002.pdf]

PLOS ONE

Doppler-Defined Pulmonary Hypertension in β-Thalassemia Major in Kurdistan, Iraq

--Manuscript Draft--

**Manuscript Number:**	PONE-D-20-17752
**Article Type:**	Research Article
**Full Title:**	Doppler-Defined Pulmonary Hypertension in β-Thalassemia Major in Kurdistan, Iraq
**Short Title:**	Pulmonary Hypertension in Thalassemia Major
**Corresponding Author:**	Ameen M Mohammad<br>University of Duhok<br>Duhok, IRAQ
**Keywords:**	Pulmonary hypertension, Thalassemia major, Iraq
**Abstract:**	Objective: Cardiopulmonary complications are among the most important complications of thalassemia major. Pulmonary hypertension is among these complications and studies addressing its frequency and associations in the latter disorder are sparse from Iraq.<br>Methods: During the period between September 2015 and February 2016, a total 100 thalassemia major patients (≥ 8 years old) were enrolled from a main thalassemia center in Kurdistan, Northern Iraq. All patients had a full history and clinical examination. Full blood count, biochemical tests and viral screen including hepatitis B surface antigen and hepatitis C virus antibody, in addition to transthoracic doppler echocardiography for tricuspid regurgitation jet velocity (TRV).<br>Results: The enrolled patients had a mean (SD) age of 17.6 (5.5) years, and included 52 males and 48 females. Pulmonary hypertension as defined as TRV> 2.8 m/s coupled with both exertional dyspnea and an absence of left sided heart failure, was identified in nine patients (9%). The latter subgroup of patients had significantly higher reticulocyte counts, S. LDH, S. ferritin, and hepatitis C seropositivity compared to those without this complication by univariate analysis. While by multivariate logistic regression only reticulocytes and hepatitis C positivity remained significant. Furthermore, TRV as a continuous variable was positively correlated with reticulocytes, S. bilirubin and LDH ($p<0.001$, 0.002 and <0.001 respectively), but not with age or S. ferritin ($P=0.77$, and 0.93 respectively).<br>Conclusion: Pulmonary hypertension is not uncommon in Iraqi patients with thalassemia major, and it appears to be linked to chronic hemolysis rather than iron overload.
**Order of Authors:**	Ameen M Mohammad
	Mohammed M Dawad
	Muna A Kashmoola
	Nasir Al-Allawi
**Additional Information:**	
**Question**	**Response**
**Financial Disclosure**<br><br>Enter a financial disclosure statement that describes the sources of funding for the work included in this submission. Review the submission guidelines for detailed requirements. View published research articles from *PLOS ONE* for specific examples.<br><br>This statement is required for submission	The author(s) received no specific funding for this work

and **will appear in the published article** if the submission is accepted. Please make sure it is accurate.<br><br>**Unfunded studies**<br>Enter: *The author(s) received no specific funding for this work.*<br><br>**Funded studies**<br>Enter a statement with the following details:<br>• Initials of the authors who received each award<br>• Grant numbers awarded to each author<br>• The full name of each funder<br>• URL of each funder website<br>• Did the sponsors or funders play any role in the study design, data collection and analysis, decision to publish, or preparation of the manuscript?<br>• **NO** - Include this sentence at the end of your statement: *The funders had no role in study design, data collection and analysis, decision to publish, or preparation of the manuscript.*<br>• **YES** - Specify the role(s) played.<br><br>* typeset	
**Competing Interests**<br><br>Use the instructions below to enter a competing interest statement for this submission. On behalf of all authors, disclose any competing interests that could be perceived to bias this work—acknowledging all financial support and any other relevant financial or non-financial competing interests.<br><br>This statement **will appear in the published article** if the submission is accepted. Please make sure it is accurate. View published research articles from *PLOS ONE* for specific examples.	The authors have declared that no competing interests exist

**NO authors have competing interests**<br><br>Enter: *The authors have declared that no competing interests exist.*<br><br>**Authors with competing interests**<br><br>Enter competing interest details beginning with this statement:<br><br>*I have read the journal's policy and the authors of this manuscript have the following competing interests: [insert competing interests here]*<br><br>* typeset	
**Ethics Statement**<br><br>Enter an ethics statement for this submission. This statement is required if the study involved:<br><br>• Human participants<br>• Human specimens or tissue<br>• Vertebrate animals or cephalopods<br>• Vertebrate embryos or tissues<br>• Field research<br><br>Write "N/A" if the submission does not require an ethics statement.<br><br>General guidance is provided below. Consult the submission guidelines for detailed instructions. **Make sure that all information entered here is included in the Methods section of the manuscript.**	"N/A"

Powered by Editorial Manager® and ProduXion Manager® from Aries Systems Corporation

**Format for specific study types**<br><br>**Human Subject Research (involving human participants and/or tissue)**<br>• Give the name of the institutional review board or ethics committee that approved the study<br>• Include the approval number and/or a statement indicating approval of this research<br>• Indicate the form of consent obtained (written/oral) or the reason that consent was not obtained (e.g. the data were analyzed anonymously)<br><br>**Animal Research (involving vertebrate animals, embryos or tissues)**<br>• Provide the name of the Institutional Animal Care and Use Committee (IACUC) or other relevant ethics board that reviewed the study protocol, and indicate whether they approved this research or granted a formal waiver of ethical approval<br>• Include an approval number if one was obtained<br>• If the study involved *non-human primates*, add *additional details* about animal welfare and steps taken to ameliorate suffering<br>• If anesthesia, euthanasia, or any kind of animal sacrifice is part of the study, include briefly which substances and/or methods were applied<br><br>**Field Research**<br><br>Include the following details if this study involves the collection of plant, animal, or other materials from a natural setting:<br>• Field permit number<br>• Name of the institution or relevant body that granted permission	
**Data Availability**<br><br>Authors are required to make all data underlying the findings described fully available, without restriction, and from the time of publication. PLOS allows rare exceptions to address legal and ethical concerns. See the PLOS Data Policy and FAQ for detailed information.	Yes - all data are fully available without restriction

A Data Availability Statement describing where the data can be found is required at submission. Your answers to this question constitute the Data Availability Statement and **will be published in the article**, if accepted.<br><br>**Important:** Stating 'data available on request from the author' is not sufficient. If your data are only available upon request, select 'No' for the first question and explain your exceptional situation in the text box.<br><br>Do the authors confirm that all data underlying the findings described in their manuscript are fully available without restriction?	
**Describe where the data may be found in full sentences. If you are copying our sample text, replace any instances of XXX with the appropriate details.**<br><br>• If the data are **held or will be held in a public repository**, include URLs, accession numbers or DOIs. If this information will only be available after acceptance, indicate this by ticking the box below. For example: *All XXX files are available from the XXX database (accession number(s) XXX, XXX.).*<br>• If the data are all contained **within the manuscript and/or Supporting Information files**, enter the following: *All relevant data are within the manuscript and its Supporting Information files.*<br>• If neither of these applies but you are able to provide **details of access elsewhere**, with or without limitations, please do so. For example:<br><br>*Data cannot be shared publicly because of [XXX]. Data are available from the XXX Institutional Data Access / Ethics Committee (contact via XXX) for researchers who meet the criteria for access to confidential data.*<br><br>*The data underlying the results presented in the study are available from (include the name of the third party*	All relevant data are within the manuscript and its Supporting Information files

and contact information or URL).<br>• This text is appropriate if the data are owned by a third party and authors do not have permission to share the data.<br><br>* typeset	
Additional data availability information:	Tick here if your circumstances are not covered by the questions above and you need the journal’s help to make your data available.

Powered by Editorial Manager® and ProduXion Manager® from Aries Systems Corporation

Doppler-Defined Pulmonary Hypertension in β-Thalassemia Major in Kurdistan, Iraq

Ameen M. Mohammad[1*] ¶, Mohammed M. Dawad,[2&] Muna A.Kashmoola,[3&] Nasir Al-Allawi[4] ¶,

1. Department of Internal Medicine, College of Medicine, University of Duhok, Duhok, Iraq.
2. Department of Hematology,Alkhansa Teaching Hospital, Mousel, Iraq.
3. Department of Pathology, College of Medicine, University of Mousel, Mousel, Iraq.
4. Department of Pathology, College of Medicine, University of Duhok, Duhok, Iraq.

Correspondence author:

Email: doctoramb@yahoo.com. (AMM)

¶These authors contributed equally to this work.

&These authors also contributed equally to this work

Abstract

Objective: Cardiopulmonary complications are among the most important complications of thalassemia major. Pulmonary hypertension is among these complications and studies addressing its frequency and associations in the latter disorder are sparse from Iraq.

Methods: During the period between September 2015 and February 2016, a total 100 thalassemia major patients (≥ 8 years old) were enrolled from a main thalassemia center in Kurdistan, Northern Iraq. All patients had a full history and clinical examination. Full blood count, biochemical tests and viral screen including hepatitis B surface antigen and hepatitis C virus antibody, in addition to transthoracic doppler echocardiography for tricuspid regurgitation jet velocity (TRV).

Results: The enrolled patients had a mean (SD) age of 17.6 (5.5) years, and included 52 males and 48 females. Pulmonary hypertension as defined as TRV> 2.8 m/s coupled with both exertional dyspnea and an absence of left sided heart failure, was identified in nine patients (9%). The latter subgroup of patients had significantly higher reticulocyte ~~counts~~, ~~S.~~ LDH, ~~S.~~ ferritin, and hepatitis C seropositivity compared to those without this complication by univariate analysis. While by multivariate logistic regression only reticulocytes and hepatitis C positivity remained significant. Furthermore, TRV as a continuous variable was positively correlated with reticulocytes, ~~S.~~ bilirubin and LDH ($p<0.001$, 0.002 and <0.001 respectively), but not with age or ~~S~~. ferritin (P=0.77, and 0.93 respectively).

Conclusion: Pulmonary hypertension is not uncommon in Iraqi patients with thalassemia major, and it appears to be linked to chronic hemolysis rather than iron overload.

Keywords: Pulmonary hypertension, Thalassemia major, Iraq

Introduction:

β-thalassemia is an autosomal recessive disorder associated with defective synthesis of β-globin chains of hemoglobin [1]. It is not only common in the Mediterranean region, South-East Asia, the Indian subcontinent and the Middle East but have now become a global problem, spreading through migration worldwide [2,3]. β-thalassemia, is associated with a variety of phenotypes ranging from severe transfusion-dependent beta thalassemia major (TM), to asymptomatic β-thalassemia minor. Pulmonary hypertension (PH) is one of the complications that have attracted increased interest recently, particularly as improved accessibility to safe blood transfusions, effective and available iron chelating medications, and the introduction of new and non-invasive methods of tissue iron assessment have led to prolonged survival [4]. The development of pulmonary hypertension in thalassemia may be the result of a multitude of pathogenic mechanisms; including chronic hemolysis, iron overload and hypercoagulability. One important consequence of chronic hemolysis and erythrocyte dysfunction is abnormal arginine –nitric oxide bioavailability [5,6]. The latter may contribute to activation of platelets, endothelial dysfunction and increase oxidative stress leading to tissue damage throughout the cardiovascular system [7].

Previous studies from Iraqi Kurdistan have looked at the frequency and associations of pulmonary hypertension in patients with sickle cell anemia and thalassemia intermedia[8,9], while reports on PH in TM are sparse. Accordingly, this study was initiated aiming to address the latter, through studying a group of thalassemia major patients attending a main thalassemia center in this part of the country.

Patients and Methods:

Patients: A total of 100 consecutive β-thalassemia major patients who were eight years or older, visiting Jin Pediatric Thalassemia Center in Duhok, Kurdistan, Iraq ~~during period~~ between September 2015 and February 2016, were recruited prior to their next scheduled transfusion.

Clinical and Laboratory assessment: The patients were clinically re-evaluated ,and their records rechecked at the time of enrollment. Their current treatment, including chelation therapy, was carefully reviewed. Blood samples were taken for the following hematological, biochemical, and serological investigations: Complete blood count (CBC), reticulocyte ~~count~~ (~~%~~), serum ferritin, liver and renal function tests, serum lactic dehydrogenase (LDH), hepatitis B surface antigen (HBsAg) and hepatitis C antibody ;using (second-generation Cobas c501 Biochemistry auto-analyzer, Roche, HITACHI,Japan).

Transthoracic Echocardiography: Standardized transthoracic echocardiographic (TTE) examination and Doppler study were performed for all cases in the Echocardiography Laboratory of Azadi Teaching Hospital, Duhok, Iraq by ~~(~~Philips Hd5 Color Doppler Ultrasound Machine, Philips Medical Systems, Netherlands~~)~~.Tricuspid valve regurgitation was measured in the apical four chambers, with parasternal short axis views, and a minimum of three sequential spectra. Pulmonary hypertension for the purposes of this study was defined as Tricuspid Regurgitant Jet velocity (TRV) in excess of 2.8 m/s coupled with both exertional dyspnea, and an absence of left heart failure [10]. Furthermore, in addition to measuring the left ventricular ejection fraction (LVEF%), the diastolic function of the left ventricle was checked by pulsed Doppler-derived left ventricle diastolic mitral inflow in the apical four-chamber view, to measure the E and A peak velocities and their ratio (E/A). All echocardiographic measurements were done according to the American Society of Echocardiography recommendations [11]. Heart failure was diagnosed based on Framingham criteria [12].

Ethical consideration: Informed consent was obtained from all participants or their guardians upon enrollment. The study was approved by the Ethics and Scientific Committee of the Iraqi Board of Medical Specialization, Baghdad, Iraq.

Statistical analysis: All statistical analyses were performed using SPSS software (release 20; SPSS inc, Chicago, IL, USA). Fisher's exact and Chi-square tests were used to compare categorical variables. Student t-test of independent samples was used for univariate analysis of continuous variables. Multivariate binary logistic regression was used for multivariate analysis. Pearson's correlation was used to assess the correlation between two continuous variables. All tests were two-sided, with a 0.05 level of significance.

Results

The mean age of the enrolled patients was 17.6, with a standard deviation (SD) of 5.5 years; and included 52 males and 48 females (M:F ratio of 1.1:1). All patients were on regular transfusions, with a mean annual number of transfusions of 16.8 units (SD =2.3). Ninety two patients were on iron chelation therapy. The latter included 59 on oral deferasirox, 19 on subcutaneous deferoxamine, and 14 on combination of the two chelators. Sixty patients (60%) were splenectomized. Hepatomegaly was encountered in 16 patients (16%). The main laboratory characteristics at the time of enrollment are outlined in Table 1.

Pulmonary hypertension ~~(PH)~~ per the definition adopted by this study was found in 9 patients (9%) [including 3 patients whose TRV≥ 3.2 m/s]. Moreover, TRV was borderline (>2.5-≤2.8 m/sec) in another 16 (16%).Among PH group: reticulocyte ~~counts~~ (%), LDH, HCV sero-positivity, and serum ferritin were significantly higher than the non-PH group by univariate analysis. There were notably higher proportions of splenectomized patients and of females in the PH subgroup, although these did not reach significance in either (Table 2). Multivariate logistic regression to include those variables with a $p \leq 0.1$, demonstrated that only reticulocyte ~~count~~ and HCV positivity remained significantly higher (Table 3). Left ventricular systolic dysfunction (as defined by LVEF% <50%) was identified in three patients in the non-PH group. All the three patients satisfied the Framingham criteria for diagnosis of heart failure.

When age, age at starting transfusions, serum ferritin, reticulocyte ~~count~~ (%), serum bilirubin, LVEF%, E/A and serum LDH correlation with TRV as continuous variables were assessed using Pearson correlation, none except ~~S.~~ LDH, serum bilirubin and reticulocyte ~~count~~ (%) showed a significant positive correlation (P=<0.001,0.002, and <0.001 respectively) [Figures 1,2; Table 4]. ~~Furthermore, TRV means were higher among females and a those splenectomized compared to males and non-splenectomized, respectively, although these were not significant (p=0.497 and 0.703, respectively).~~

Figure 1. Scatter plot showing the correlation between TRV and LDH in100 TM patients.

Figure 2. Scatterplot showing correlation between TRV and reticulocyte count in 100 TM patients.

Discussion

While pulmonary hypertension is a well-recognized complication of thalassemia major, its doppler-defined frequencies in various TM series showed remarkable variability ranging from1.7% to 79% [13-19].The most important cause of such variability is the choice of the TVR cut-off point. While many studies used a cut- off of 2.5 m/s, others used 2.8 m/s, or higher values of 3.0 or 3.2 m/sec. The choice of a higher cut-off would ensure higher specificity, but on the expense of sensitivity [20]. Looking at our data: 25% had TVRs>2.5m/s, compared to 9% and 3% for TVRs >2.8 m/s and ≥ 3.2 m/s respectively. For the purposes of the current study we used the cut-off point of 2.8 m/s, coupled by exertional dyspnea and in the absence of left ventricular failure, to increase specificity and decrease false positive cases [10,21]. However, it is important to note that the gold standard for the diagnosis of PH is right heart catheterization (RHC), and it has been documented that despite moderate correlation between the latter and echocardiography, echocardiography tends to overestimate the actual frequency of PH [22]. However, RHC is an invasive procedure, thus the vast majority of reports, similar to ours, utilized the non-invasive echocardiography for screening for PH in hemoglobinopathies.

Another cause of variability in the frequency of PH in TM is the adequacy of management, and Aessopos et al (2005, 2004)and Meloni et al (2015) reported that in well -managed patients, PH is virtually absent, while the high prevalence rates were more likely to be reported in TM series where management was inadequate [19,23,24].Actually it has been proposed that timely transfusion and adequate chelation may delay the emergence of pulmonary hypertension in TM patients [23,24], by restoring tissue delivery of oxygen , suppressing production of defective red cells, reducing hemolysis and ameliorating hypercoagulability [25]. Among our patients, the management as judged by pre-transfusion hemoglobin and the mean serum ferritin seems to be less than optimal, since pre-transfusion hemoglobin was more than 9 g/dl in only 41%, compared to nearly 90% in some studies from developed countries, while our mean serum ferritin was

evidently higher than reported from the latter countries[26,27].Another cause of variation in the reported rates of PH among thalassemia major patients is the age range of the recruited patients, and studies including mainly adults would be expected to have higher rates, than those focusing mainly on children. In this series we did not include children less than 8 years old, since PH is unlikely and we enrolled patients older than this age, with our youngest patient diagnosed as PH being 10 years old, which is consistent with the literature [17].

Pulmonary hypertension is more likely to occur among thalassemia intermedia ~~(TI)~~ than TM, and our rate of 9% is less than the 20.4% reported in an earlier study from our center on TI using the same diagnostic criteria for PH [9]. The lower frequency of PH among TM, compared to intermedia, has also been documented by many previous studies worldwide [18,24,28]. It appears that more frequent and regular transfusion in TM may be the main culprit of this difference. In the earlier study on TI at our center, 83.8% were sparsely or rarely transfused [9]. This notion may be at the center of the argument of the need to transfuse patients with non-transfusion dependent thalassemia (NTDT) more regularly to avoid or delay PH [28].

Female predominance, though insignificant, among patients with PH in the current study is shared by some reports [29,30], but disputed by others [17-19].Strong female predominance in many forms of PH is well recognized [31]. This has been ascribed to altered estrogen levels and metabolism in the females, which affect the pulmonary vasculature and the right ventricle in a way that predisposes to PH [32].

A significant association of PH with hepatitis C seropositivity, as detected in the current study, is matched by some studies on TM [17]. This observation is not unexpected since HCV infection has been linked to right ventricular dysfunction, increased pulmonary vascular resistance and pulmonary hypertension [33]. Furthermore, chronic liver diseasehas been associated with pulmonary hypertension [34,35].

Several studies have implicated splenectomy as a risk factor for PH in thalassemia [17,18,29,36]. It appears that the loss of the spleen filtering functioning may lead to increased circulating abnormal red cells, nucleated red cells, red cell breakdown products, platelet activation and pro-coagulant factors release, which may eventually lead to

increased risk of intravascular thrombosis and changes in pulmonary vasculature leading to PH[36-38].In the current study, the proportion of splenectomized patients was much higher in the PH subgroup, but did not reach significance. Such absence of significant association is not unique and has beenreported by at least one previous study [19].

The association between higher reticulocytes and LDH with PH, and between higher reticulocytes, LDH and bilirubin with TRV is interesting and likely indicates that PH (as identified by TRV >2.8 m/s) and increasing TRV (as a continuous variable regardless of any cut-off points) are linked to chronic hemolysis. Hemolysis would lead to release of cell-free hemoglobin and arginase and thus lead to impaired nitric oxide availability and endothelial dysfunction and eventually pulmonary hypertension, and this has documented in both sickle cell anemia and thalassemia [39,40].

Although serum ferritin was significantly higher among PH patients by univariate analysis, this did not remain so in the multivariate one. Similarly we failed to find an association between S. ferritin and TRV. This favors the conclusion that among our patients and in consistence with some earlier studies, chronic hemolysis, rather than iron overload, plays a major role in the pathogenesis of PH in our TM patients [29,30,41].This is consistent with the notion that among the less than adequately transfused/chelated patients, hemolysis rather than iron overload is the main culprit of PH [5].

The importance of early identification of PH in thalassemia should be underscored, since it is associated with right ventricular dysfunction and failure and increased mortality [42]. While a variety of management options have been tried, ranging from the institution of more adequate transfusion and chelation, to hydroxycarbamide, L-carnitine, phosphodiesterasetype 5 inhibitors, endothelial receptor antagonists and synthetic prostacyclins, however these therapeutic option are still waiting validation by double blind trials[5,43].

Strengths and limitations

The main limitation of the current study is that it did not include using the invasive RHC to confirm PH, though the use of TRV cut off point of 2.8 m/s coupled by clinical

findings was shown by earlier studies to correlate well with RHC. Another limitation is relevant to the study being a cross sectional study, so it did not include follow up or response to management, and we hope that future prospective studies will address these aspects. On the other hand, the strength of our study is that it showed that PH as defined by a TRV cut off point, and TRV as a continuous variable were both correlated to markers of hemolysis.

Conclusions

In this study we found that the PH is not uncommon, and is often missed, among Iraqi patients with TM and its pathogenesis is more closely linked to chronic hemolysis than to iron overload. Early screening by echocardiography starting at the age of 10 years, followed by confirmation by RHC in selected cases should be underscored. The importance of timely transfusion and adequate chelation is of paramount importance. Moreover, it may be worthwhile to consider initiating a multicenter study to assess the value of various proposed therapeutic options in this part of the world.

Competing Interests

We have read the journal's policy and the authors of this manuscript have no competing interests

Funding

The authors received no specific funding for this research.

Acknowledgments

We acknowledge the cooperation of Dr. Saad Younis, the epidemiologist in the University of Duhok, the staff of echocardiograpgy laboratory with staff of thalassemia center and the enrolled patients and their families.

Author Contributions

Conceptualization: AMM NA MAK.

Data curation: MMD AMM.

Formal analysis: NA AMM MAK MMD.

Investigation: AMM MMD NA.

Methodology: AMM NA MMD MAK.

Project administration: MAK NA AMM.

Resources: MMD AMM.

Supervision: AMM NA MAK.

Validation: AMM NA MAK MMD.

Visualization: NA AMM MAK MMD.

Writing – original draft: AMM NA MMD MAK.

Writing – review & editing: NA AMM MAK.

References

1. Rachmilewitz EA,Giardina PJ (2011) How I treat thalassemia. Blood 118: 3479-3488.
2. Colah R, Gorakshakar A, Nadkarni A (2010) Global burden, distribution and prevention of β-thalassemias and hemoglobin E disorders. Expert Rev Hematol 3(1):103-117.
3. Sayani FA, Kwiatkowski JL (2015) Increasing prevalence of thalassemia in America: Implications for primary care. Ann Med47(7):592-604
4. Amid A, Saliba AN, Taher AT, Klaassen RJ (2015)Thalassaemia in children: From quality of care to quality of life. Arch Dis Child 100(11):1051-7
5. FraidenburgDR, Machado RF (2016)Pulmonary hypertension associated with thalassemia syndromes. Ann NY AcadSci 1368(1): 127-139.
6. Morris CR, Vinchinsky EP (2010)Pulmonary hypertension in thalassemia. Ann. NY AcadSci 1210(1): 205-213.
7. Villagra J, Shiva S, Hunter LA, Machado RF, Gladwin MT, Kato GJ (2007)Platelet activation in patients with sickle cell disease, hemolysis associated pulmonary hypertension, and nitric oxide scavenging by cell-free hemoglobin. Blood 110: 2166-72.
8. Al-Allawi N, Mohammad AM, Jamal S (2016) Doppler-Define Pulmonary Hypertension in Sickle cell Anemia in Kurdistan, Iraq. *Plos One*: 11(9): e0162036.doi:10.1371/journal.pone.0162

9. Al-Allawi NA, Jalal SD, Mohammad AM, Omer SQ, Markous RS (2014) β-thalassemia Intermedia in Northern Iraq: A single center experience. BioMed Research International; 2014:262853. doi: 10.1155/2014/262853.

10. Barst RJ, McGoon M, Toricki A, Sitbon O, Krowka MJ, Olschewski H, Gaine S (2004) Diagnosis and differential assessment of pulmonary arterial hypertension. J. AmerCollCardiol 43(12Suppl S): 40S-47S.

11. Masuyama T, Kodama K, Kitabatake A, Sato H, Nanto S, Inoue M (2006) Continuous-wave Doppler echocardiographic detection of pulmonary regurgitation and its application to noninvasive estimation of pulmonary artery pressure. Circulation. 74(3):484–492.

12. Ho KK, Pinsky JL, Kannel WB, Levy D (1993)The epidemiology of heart failure: The Farmingham study. J. Am. Coll. Cardiol22(4 suppl A): 6A-13A.

13. Du ZD, Roguin N, Milgram E, Saab K, Koren A(1997) Pulmonary hypertension in patients with thalassemia major. Am Heart J 134:532-7.

14. Derchi G, Fonti A, ForniGL ,Galliera EO, Cappellini MD, Turati F,Policlinico OM (1999) Pulmonary hypertension in patients with thalassemia major. Am Heart J 138:384

15. Hagar RW, Morris CR, Vichinsky EP (2006)Pulmonary hypertension in thalassemia major patients with normal left ventricular systolic function. Brit. J Hematol 133; 433-435.

16. Kiter G, Balci YI, Ates A, Hacioglu S, Sari I (2010) Frequency of pulmonary hypertension in asymptomatic beta-thalassemia major patients and the role of physiological parameters in evaluation. PediatrHematolOncol 27(8): 597-607.

17. Morris CR, Kim HY, Trachtenberg F, Wood J, Quinn C, Sweeters N, Kwaitkowski JL, Thompson AA, Giardina PJ, Boudreaux J,Olivieri NF, Porter JB,Neufeld EJ, Vichinsky EP (2011) Risk factors and mortality associated with an elevated tricuspid regurgitant jet velocity measured by Doppler-echocardiography in thalassemia: a Thalassemia Clinical Research Network report. Blood118(14):3794–3802.

18. Derchi G, Galanello R, Bina P, Cappellini MD, Piga A, Lai ME, Quarta A, Casu G, Perrotta S, Pinto V, Musallam KM, Forni GL (2014) Prevalence and risk factors for pulmonary arterial hypertension in a large group of β-thalassemia patients using right heart catheterization: a Webthalstudy.Circulation129(3):338-45.

19. Meloni A, Detterich J, PepeA,Harmatz P, Coates TD, Wood JC (2015) Pulmonary hypertension in well-transfused thalassemia major patients. Blood Cells Mol Dis 54(2): 189-194.

20. Galié N, Hoeper MM, Humbert M, Torbicki A, Vachiery JL, Barbera JA, Beghetti M, Corris P, Gaine S, Gibbs JS, Gomez-Sanchez MA, Jondeau G, Klepetko W, Opitz C, Peacock A, Rubin L, Zellweger M, Simonneau G; ESC Committee for Practice Guidelines (CPG)(2009) Guidelines for the diagnosis and treatment of pulmonary hypertension of the European society of Cardiology (ESC) and the European respiratory society (ERS), endorsed by the international society of heart and lung transplantation (ISHLT). Eur Heart J 30 (20):2493-2537.

21. Taher AT, Musallam KM, Karimi M,El-Beshlawy A, Belhoul K, Daar S, Saned M-S, El-Chafic A-H, Fasulo MR, Cappellini MD (2010) Overview on practices in thalassemia intermedia management aiming for lowering complication rates across region of endemicity: the optimal care study. Blood 115(10): 1886-1892.

22. Janda S, Shahidi N, Gin K, Swiston J (2011) Diagnostic accuracy of echocardiography for pulmonary hypertension: a systematic review and meta-analysis. Heart97:612-622.

23. Aessopos A, Farmakis D, Hatziliami A, Fragodimitri C, Karabatsos F, Joussef J, Mitilineou E, Diamanti-Kandaraki E, Meletis J, Karagiorga M (2004) Cardiac status in well-treated patients with thalassemia major. Europ J Hematol 73:359-366.

24. Aessopos A, Farmakis D (2005)Pulmonary hypertension in beta-thalassemia. Ann NY AcadSci 1054: 342-349.

25. Machado RF, Farber HW (2013) Pulmonary Hypertension Associated with Chronic Hemolytic Anemia and Other Blood Disorders. Clin Chest Med.; 34(4): 739–752.

26. Cunningham MJ, Macklin EA, Neifeld EJ, Cohen AR (2004) Thalassemia Clinical Research Network. Complications of beta-thalassemia major in North America. Blood 104(1): 34-9.

27. Thuret I, Pondarré C, Loundou A, Steschenko D, Girot R, Bachir D, Rose C, Barlogis V, Donadieu J, de Montalembert M, Hagege I, Pegourie B, Berger C, Micheau M, Bernaudin F, Leblanc T, Lutz L, Galactéros F, Siméoni M-C, Badens C(2010) Complications and treatment of patients with β-thalassemia in France: results of the national registry. Haematologica 95(5): 724-9.

28. Karimi M, Musallam KM, Cappellini MD, Darr S, Elbashelawy A, Belhoul K, Saned MS, Temraz S, Koussa S, Taher AT(2011) Risk factors for

pulmonary hypertension in patients with β-thalassaemiaintermedia. Eur J Intern Med 22: 607-610.

29. Atichartakarn V, Likittanasombat K, Chuncharunee S, Chandanamattha P WorapongpaiboonS,Angchaisuksiri P,Aryurachai K (2003) Pulmonary arterial hypertension in previously splenectomizedpatients with β-thalassemic disorders.Int. JHematol78(2): 139-145.

30. Amoozgar H, Farhani N, Karimi M (2010) Risk factors for pulmonary hypertension in patients with thalassemia intermedia. Eur J Haematol 85: 549-551.

31. Badesch DB, Raskob GE, Elliot CG, Krichman AM, Farber HW, Frost AE, Barst RJ, Benza RL, Liou TG, Turner M, Giles S, Feldkircher K, Miller DP, McGoon MD. (2010)Pulmonary arterial hypertension: baseline characteristics from the REVEAL registry. Chest 137(2): 376-387.

32. Pugh ME, HemnesAR (2010). Pulmonary hypertension in women. Expert Rev CardiovascTher 8 (11): 1549-1558.

33. DemirC,Demir M (2015) Effect of hepatitis C virus infection on the right ventricular function, pulmonary artery pressure and pulmonary vascular resistance. Biomedical Research 26(1): 183-187.

34. Mandell MS, Groves BM (1996)Pulmonary hypertension in chronic liver disease. Clin Chest Med 17;17.

35. Silva SS (2000). Liver disease and pulmonary hypertension. Gut; 47(4):595.

36. Phrommintikul A, Sukonthasarn A, Kanjanavanit R, Nawarawong W (2006)Splenectomy : a strong risk factor for pulmonary hypertension in patients with thalassaemia. Heart 92: 1467-72.

37. Ruf A,Pick M, Deutsch V, Patscheke H, Goldfarb A, Rachmilewitz EA, Guillin MC, Eldor A(1997)Invivo platelet activation correlates with red cell anionic phospholipid exposure in patients with beta-thalassemia major. Brit J Haematol98: 51-56.

38. Eldor A, Rachmilewitz EA (2002)Thehypercoagulable state in thalassemia. Blood 99: 36-43.

39. Morris CR, Kuypers FA, Kato GJ, Lavrisha L, Larkin S, Singer T, Vichinsky EP (2005) Hemolysis-associated pulmonary hypertension in thalassemia. Ann NY AcadSci 1054: 481-5.

40. Reiter CD, Wang X, Tanus-Santos JE, Hogg N, Cannon RO, Schechter AN, Gladwin MT (2002) Cell-free hemoglobin limits NO bioavailability in Sickle cell disease. Nature Med 8: 1383-1389.

41. Singer ST, Kuypers FA, Styles l, Vichinsky EP, Foote D, Rosenfeld H.(2006) Pulmonary hypertension in thalassemia: association with platelet activation and hypercoagulable state. Am J Hematol81(9):670-675.

42. Benza RL,Miller DP, Barst RJ, Badesch DB, Frost AE, McGoon MD (2012)An evaluation of long-term survival from the time of diagnosis in pulmonary arterial hypertension from the REVEAL registry. Chest 142: 448-56.

43. Taher AT, Cappellini MD (2018) How I manage medical complications of β-thalassemia in adults. Blood 132 (17): 1781-91.

Table 1.Main laboratory findings among 100 thalassemia major patients at enrollment.

Parameter	Mean (SD)
Hemoglobin (g/dL)	8.6 (1.16)
WBC (x 10^9/L)	18.4 (14.5)
Platelets (x 10^9/L)	360 (201)
Reticulocyte count (%)	5.0 (2.2)
S. ALT (IU/L)	73.7(52.1)
S. AST (IU/L)	72.1 (51.3)
S. Bilirubin (mg/dL)	1.75 (0.97)
S. Urea (mg/dl)	30.6 (12.2)
S. Creatinine (mg/dl)	0.49 (0.15)
S. Ferritin (ug/L)	3903 (2641)
S. LDH (U/L)	303.8 (150.1)
HBsAg	5 (5%)*
HCV Antibody	25 (25%)*

Results are mean (standard deviation), *number (percentage). WBC: White blood cell, S. ALT: serum alanaine transaminase, S. AST: serum Asparatate transaminase, S. LDH: serum lactate dehyrogenase, HBsAG: hepatitis B surface antigen, HCV: hepatitis C virus

.

Table 2.Comparison between various parameters in patients with TRV> 2.8 and those with TRV ≤ 2.8 m/s.

Parameter	TRV>2.8 m/s	TRV≤ 2.8 m/s	Univariate
Age (years)	18.6 ± 4.6	17.5±5.6	0.59
Sex	2 M/7 F	50 M/41 F	0.127
Age of starting transfusions (months)	8.1 ± 2.6	7.2 ± 2.4	0.296
Annual rate of Transfusions	18.1±1.9	16.6 ± 2.3	0.063
Splenectomy	7 (77.8%)	53 (58.2%)	0.433
Hepatomegaly	1 (11.1%)	15 (16.5%)	0.95
Hb (g/dL)	8.6 ±1.1	8.7± 1.2	0.85
WBC (x109/L)	22.2 ± 19.3	18.0 ± 14	0.41
Platelets (x109/L)	392 ± 219	357 ± 200	0.62
Reticulocyte count (%)	7.50 ±1.1	4.77 ± 2.1	<0.001
ALT (IU/L)	93.4 ± 41.6	71.79 ± 52.82	0.236
AST (IU/L)	85.1 ±36.1	70.79 ± 52.53	0.427
Bilirubin (mg/dL)	1.99 ±0.96	1.64 ± 0.82	0.236
Urea (mg/dl)	27.9 ±5.06	30.91 ± 12.64	0.480
Creatinine (mg/dl)	0.45 ± 0.15	0.50 ± 0.15	0.333
S. Ferritin (ug/L)	5603 ±3172	3735 ± 2542	0.042
LDH (U/L)	520.1 ±224.7	282.4 ± 123.1	<0.001
HCVsero-positivity	8 (88.9%)	17 (18.6%)	<0.001
HBsAg detected	1 (11.1%)	4 (4.4%)	0.765
LVEF (%)	58.8 ±5.9	62.1 ± 6.0	0.117
E/A	1.71 ± 0.32	1.76 ± 0.24	0.557

HB: hemoglobin, WBC: White blood cell, S. ALT: serum alanaine transaminase, S. AST: serum Asparatate transaminase, S. LDH: serum lactate dehyrogenase, HBsAG: hepatitis B surface antigen, HCV: hepatitis C virus, LVEF: left ventricle ejection fraction, E/A: ratio for diastolic dysfunction

Table 3. Multivariate analysis to compare between those with TRV >2.8 and those with TRV ≤ 2.8 m/s on parameters with a P value <0.1 by univariate analysis.

Parameter	B	S.E.	Wald	P value.	Odds Ratio	95% Confidence Interval OR
Reticulocyte count	.709	.347	4.169	.041	2.031	1.029-4.010
S. LDH	.003	.003	1.333	.248	1.003	.998-1.009
S. Ferritin	.000	.000	1.383	.240	1.000	1.000-1.001
Annual Transfusion Frequency	.413	.310	1.774	.183	1.512	.823-2.778
HCV Positivity	3.273	1.278	6.560	.010	26.402	2.157-323.214

Table 4.Correlation between continuous variables with TRV.

Parameter	Pearson correlation coefficient	P-value
Age	0.029	0.774
Age at starting transfusions	-0.054	0.595
Annual Transfusion frequency	0.124	0.160
Hemoglobin	-0.129	0.20
Reticulocyte ~~count~~	0.149	<0.001
Bilirubin	0.307	0.002
LDH	0.623	<0.001
Leucocyte count	-0.009	0.93
Platelet count	0.002	0.986
~~S.~~ Ferritin	0.009	0.160
Ejection fraction (LVEF%)	-0.153	0.129
E/A	-0.018	0.855

Figure Click here to download Figure Fig 1,2.docx

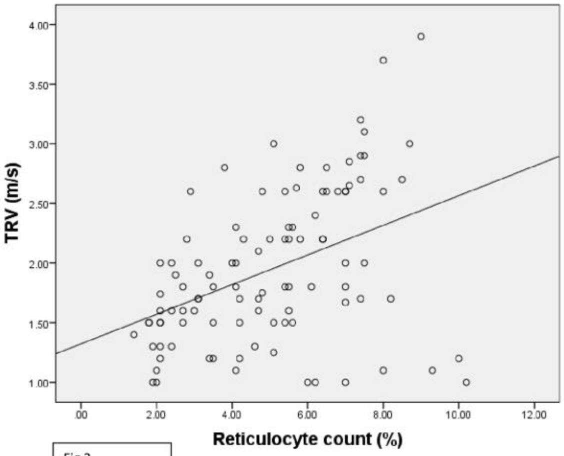

Fig 2

fFigf

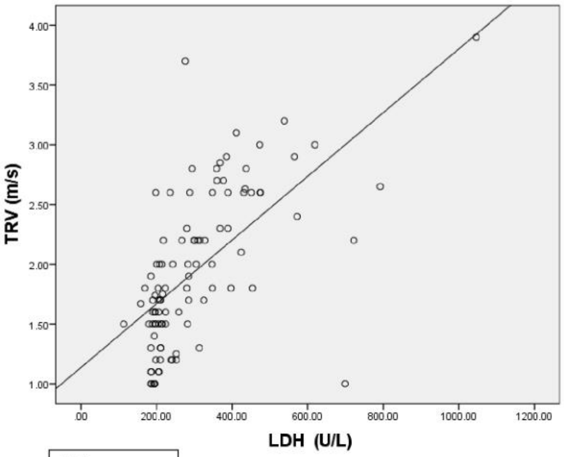
4.00
3.50
3.00
2.50
2.00
1.50
1.00
TRV (m/s)
.00
200.00
400.00
600.00
800.00
1000.00
1200.00
LDH (U/L)

Fig 1
