# Supplementary material for: Doppler-defined pulmonary hypertension in β-thalassemia major in Kurdistan, Iraq
Source: PLoS One. 2020 Dec 10;15(12):e0243648. doi: 10.1371/journal.pone.0243648 (PMC7728223; doi:10.1371/journal.pone.0243648)
Supplement: Attachment — Submitted filename: Response to Editors and reviewers comments.docx [file pone.0243648.s003.docx]

***RESPONSE TO EDITORS & REVIEWERS***

Journal Requirements:

When submitting your revision, we need you to address these additional requirements.

Style requirements for title page, abstract, references were followed and appropriate corrections were made where needed.

2. PLOS requires an ORCID iD for the corresponding author in Editorial Manager on papers submitted after December 6th, 2016. Please ensure that you have an ORCID iD and that it is validated in Editorial Manager. To do this, go to ‘Update my Information’ (in the upper left-hand corner of the main menu), and click on the Fetch/Validate link next to the ORCID field. This will take you to the ORCID site and allow you to create a new iD or authenticate a pre-existing iD in Editorial Manager. Please see the following video for instructions on linking an ORCID iD to your Editorial Manager account: https://www.youtube.com/watch?v=_xcclfuvtxQ

Added at the appropriate site

[Note: HTML markup is below. Please do not edit.]

Reviewers' comments:

Reviewer's Responses to Questions

**Comments to the Author**

1. Is the manuscript technically sound, and do the data support the conclusions?

The manuscript must describe a technically sound piece of scientific research with data that supports the conclusions. Experiments must have been conducted rigorously, with appropriate controls, replication, and sample sizes. The conclusions must be drawn appropriately based on the data presented.

Reviewer #1: Yes

Reviewer #2: Yes

2. Has the statistical analysis been performed appropriately and rigorously?

Reviewer #1: Yes

Reviewer #2: Yes

3. Have the authors made all data underlying the findings in their manuscript fully available?

The [PLOS Data policy](http://www.plosone.org/static/policies.action#sharing) requires authors to make all data underlying the findings described in their manuscript fully available without restriction, with rare exception (please refer to the Data Availability Statement in the manuscript PDF file). The data should be provided as part of the manuscript or its supporting information, or deposited to a public repository. For example, in addition to summary statistics, the data points behind means, medians and variance measures should be available. If there are restrictions on publicly sharing data—e.g. participant privacy or use of data from a third party—those must be specified.

Reviewer #1: Yes

Reviewer #2: Yes

4. Is the manuscript presented in an intelligible fashion and written in standard English?

PLOS ONE does not copyedit accepted manuscripts, so the language in submitted articles must be clear, correct, and unambiguous. Any typographical or grammatical errors should be corrected at revision, so please note any specific errors here.

Reviewer #1: Yes

Reviewer #2: Yes

5. Review Comments to the Author

Please use the space provided to explain your answers to the questions above. You may also include additional comments for the author, including concerns about dual publication, research ethics, or publication ethics. (Please upload your review as an attachment if it exceeds 20,000 characters)

Reviewer #1: The trial is important to better understand thalassemia and why is very important to make the correct treatment.
Please, inform in the introduction that PH is mainly in NTDT

A statement to this effect was introduced: lines 55-59 page 3 First paragraph introduction.

At the end of introduction, be more objective when describing the trial objective

The statement at the end of introduction was modified as requested: Lines 69-70 page 3 introduction.

PLOS uses the reference style outlined by the International Committee of Medical Journal Editors (ICMJE), also referred to as the “Vancouver” style. Please, correct the references.

The reference style used by the Journal was employed as requested pages 13-16

Please see some suggestions in the attached PDF.

Almost all suggested modifications were performed as requested by reviewer, and we are very thankful for the reviewer's constructive and in depth review.

The modifications include changing two references, and changing some statements as requested.

English could be improved.

Multiple changes were introduced as appropriate.

Reviewer #2: Despite the limitations of echocardiography to evaluate RV function, it is possible by the FAC (fractional area change) and TAPSE ( tricuspid annular plane systolic excursion). So if you have these data, I think it is important to add it to the manuscript, at least for the patients with pulmonary hypertension. It is not essential because it is not one of the objectives but it is important.

We agree with the reviewer of the additional informative value of FAC and TAPSE, unfortunately this information is not available to us to add to the results.
